# A Novel Regulatory Function of Sweet Taste-Sensing Receptor in Adipogenic Differentiation of 3T3-L1 Cells

**DOI:** 10.1371/journal.pone.0054500

**Published:** 2013-01-15

**Authors:** Yosuke Masubuchi, Yuko Nakagawa, Jinhui Ma, Tsutomu Sasaki, Tadahiro Kitamura, Yoritsuna Yamamoto, Hitoshi Kurose, Itaru Kojima, Hiroshi Shibata

**Affiliations:** 1 Department of Cell Biology, Institute for Molecular and Cellular Regulation, Gunma University, Maebashi, Japan; 2 Metabolic Signal Research Center, Institute for Molecular and Cellular Regulation, Gunma University, Maebashi, Japan; 3 Divsion of Environmental Medicine, Defense Medicine Research Institute, National Defense Medical College, Tokorozawa, Japan; 4 Department of Pharmacology and Toxicology, Graduate School of Pharmaceutical Sciences, Kyushu University, Fukuoka, Japan; Georgia Health Sciences University, United States of America

## Abstract

**Background:**

Sweet taste receptor is expressed not only in taste buds but also in nongustatory organs such as enteroendocrine cells and pancreatic beta-cells, and may play more extensive physiological roles in energy metabolism. Here we examined the expression and function of the sweet taste receptor in 3T3-L1 cells.

**Methodology/Principal Findings:**

In undifferentiated preadipocytes, both T1R2 and T1R3 were expressed very weakly, whereas the expression of T1R3 but not T1R2 was markedly up-regulated upon induction of differentiation (by 83.0 and 3.8-fold, respectively at Day 6). The α subunits of Gs (Gαs) and G14 (Gα14) but not gustducin were expressed throughout the differentiation process. The addition of sucralose or saccharin during the first 48 hours of differentiation considerably reduced the expression of peroxisome proliferator activated receptor γ (PPARγ and CCAAT/enhancer-binding protein α (C/EBPα at Day 2, the expression of aP2 at Day 4 and triglyceride accumulation at Day 6. These anti-adipogenic effects were attenuated by short hairpin RNA-mediated gene-silencing of T1R3. In addition, overexpression of the dominant-negative mutant of Gαs but not YM-254890, an inhibitor of Gα14, impeded the effects of sweeteners, suggesting a possible coupling of Gs with the putative sweet taste-sensing receptor. In agreement, sucralose and saccharin increased the cyclic AMP concentration in differentiating 3T3-L1 cells and also in HEK293 cells heterologously expressing T1R3. Furthermore, the anti-adipogenic effects of sweeteners were mimicked by Gs activation with cholera toxin but not by adenylate cyclase activation with forskolin, whereas small interfering RNA-mediated knockdown of Gαs had the opposite effects.

**Conclusions:**

3T3-L1 cells express a functional sweet taste-sensing receptor presumably as a T1R3 homomer, which mediates the anti-adipogenic signal by a Gs-dependent but cAMP-independent mechanism.

## Introduction

The sweet taste receptor expressed in taste receptor (type II) cells of taste buds consists of two members of the T1R family class C G protein-coupled receptors (GPCRs), T1R2 and T1R3 [1], that are characterized by a large extracellular venus flytrap domain (VFD) linked to a canonical 7-transmembrane domain (TMD) via a short cysteine-rich domain (CRD). This heterodimeric receptor is activated by a significant number of structurally distinct agonists, including saccharides, amino acids, sweet proteins and artificial sweeteners, with different types of compounds potentially binding to different portions of the receptor [2]. While the precise signaling mechanisms downstream of the sweet taste receptor has yet to be fully defined, one accepted signal transduction cascade is that the T1R2 and T1R3 heterodimer is coupled with gustducin, a heterotrimeric G protein expressed selectively in taste receptor cells, which activates phospholipase C-β2 (PLCβ2) resulting in the hydrolysis of phosphatidylinositol 4,5-bisphosphate into inositol 1,4,5-trisphosphate (IP_3_) and diacylglycerol (DAG). IP_3_ triggers the release of calcium from the endoplasmic reticulum with a subsequent elevation of the cytosolic calcium concentration ([Ca^2+^]_c_). This increase in [Ca^2+^]_c_ activates a non-selective cation channel, TRPM5, causing sodium ion influx and membrane depolarization, allowing release of ATP through ATP-permeable pannexin1 hemichannels. Released ATP, directly or indirectly via the stimulation of neighboring presynaptic (type III) cells, excites sensory afferent fibers. Although several lines of evidence from morphological, heterologous expression and knockout mice studies have supported this model (for review see [3]), it may not be the sole mechanism of sweet taste signal transduction. For example, mice deficient in either T1R2 or T1R3 show greatly diminished but not abolished response to some sweet compounds [4]–[6]. Additionally, gustducin or TRPM5 knockout mice are not completely unresponsive to sweet compounds [7]–[9]. These observations have suggested that other undefined sweet taste-sensing receptor(s) and signal transduction mechanisms may exist for recognition of sweet stimuli.

On the other hand, it has become evident in recent years that the sweet taste receptor is expressed not only in taste buds but also in nongustatory organs such as enteroendocrine cells [10] and pancreatic beta-cells [11]. Thus, stimulation of the sweet taste receptor in endocrine cells of the intestine causes the release of incretin hormones such as glucagon-like peptide-1 (GLP-1) and glucose-dependent insulinotropic polypeptide (GIP), which up-regulate the expression of a sodium-dependent glucose transporter, SGLT1, in enterocytes and increase the absorption of glucose from the intestinal lumen [10], [12], [13]. In pancreatic beta-cells, stimulation of the sweet taste receptor elicits insulin release by elevating [Ca^2+^]_c_ and/or [cAMP]_c_ [11]. These observations have unveiled novel nongustatory functions of the sweet taste receptor and raised a possibility that it may play more extensive roles in energy metabolism, whereas its expression and function in adipocytes have remained unknown. In the present study, we examined the expression and function of the sweet taste receptor in 3T3-L1 cells. We show here that a functional sweet taste-sensing receptor is expressed in differentiating adipocytes and plays a negative regulatory role in adipogenesis.

## Materials and Methods

### Materials

Rabbit antibodies for PPARγ, C/EBPα, and aP2/FABP4 were purchased from Cell Signaling Technologies Inc. (Danvers, MA). Guinea pig anti-GLUT4 antibody was raised in this laboratory as described previously [14]. Rabbit polyclonal anti-T1R3 antibody was purchased from Abcam (Cambridge, UK). Mouse monoclonal anti-tubulin (clone TUB 2.1) and anti-actin (clone AC-40) antibodies, sucralose and Oil red-O were obtained from Sigma (St Louis, MO). Sodium saccharin, D-mannitol and cholera toxin were from Wako Pure Chemical Industries (Osaka, Japan). Endothelin-1 was purchased from Peptide Institute, Inc. (Osaka, Japan). YM-254890, a generous gift of Jun Takasaki (Astellas Pharma Inc., Tsukuba, Japan), was dissolved in dimethyl sulfoxide at 10 mM as a stock solution. The RNAs of mouse circumvallate and foliate papillae were kindly provided by Yutaka Maruyama and Yuzuru Etoh (Ajinomoto Company, Inc., Kawasaki, Japan) [15].

### Cell Culture and Differentiation

3T3-L1 cells provided by Howard Green (Harvard Medical School, Boston, MA) [16] were maintained in Dulbecco's modified Eagle's medium containing 4.5 g/L D-glucose (DMEM-HG) supplemented with 50 μg/ml penicillin, 75 μg/ml streptomycin and 10% calf serum (CS) at 37°C in a humidified atmosphere of 5% CO_2_, and were differentiated into adipocytes as described previously [17]. Briefly, 2 days after confluence, the medium was replaced with fresh DMEM containing 1.0 g/L D-glucose (DMEM-LG) supplemented with 10% fetal bovine serum (FBS), 0.5 mM 1-methyl-3-isobutylxanthine (IBMX), 10 μM dexamethasone, and 1.7 μM insulin. Forty-eight hours later, the medium was replaced with fresh DMEM-LG containing 10% FBS and 1.7 μM insulin. After 48 hours, insulin was withdrawn from the culture media and cells were maintained in DMEM-LG containing 10% FBS.

### Animals

Male C57BL/6J mice were purchased from Clea Japan Inc. (Tokyo, Japan). They were kept in an experimental animal facility controlled at 23°C with a 12-hour light and dark cycle, and with free access to standard chow and water. The animal experiment was conducted according to the guidelines for animal care issued by the Animal Experiment and Ethic Committee, Gunma University. The protocol was approved by the Animal Experiment and Ethic Committee, Gunma University (Permit Number: 09–069). All procedures including incision in the abdomen to obtain the epididymal fat pads were performed under sodium pentobarbital anesthesia, and all efforts were made to minimize suffering.

### Preparation of Adipocyte and Stromal-Vascular Fractions

Adipocyte and non-adipocyte fractions were prepared by the collagenase digestion method [18] from the epididymal adipose tissues of C57BL/6J mice (6-week old). Briefly, the adipose tissues were digested for 45 minutes at 37°C with 2 mg/ml collagenase (Type I, from Worthington Biochemical Corporation, Lakewood, NJ) in Krebs-Henseleit Hepes buffer (118 mM NaCl, 4.74 mM KCl, 2.54 mM CaCl_2_, 1.18 mM KH_2_PO_4_, 1.18 mM MgSO_4_, 30 mM Hepes/NaOH, pH 7.4) supplemented with 40 mg/ml BSA (fraction V) and 3 mM sodium pyruvate (Buffer A), and centrifuged at 3000 rpm for 10 minutes. Floating cells and the pellet were separated, washed 4 times with Buffer A and used as mature adipocytes and the stromal-vascular fraction (SVF), respectively.

### Preparation and Differentiation of Adipose Tissue-derived Stromal Cells

Primary mouse adipose tissue-derived stromal cells (ATSCs) were prepared from epididymal adipose tissues of C57BL/6J mice (6-week old) as described previously [19] with a slight modification. Briefly, the adipose tissues were washed extensively with DMEM-LG and digested for 120 minutes at 37°C with 1 mg/ml collagenase (Type I, from Wako Pure Chemical Industries, Osaka, Japan). The cells were filtered through a 40-μm nylon mesh (Becton Dickinson, NJ), and then centrifuged at 3000 rpm for 10 min. The pellet was resuspended in DMEM-LG containing 10% FBS and antibiotics (70 μg/ml penicillin and 100 μg/ml streptomycin) and cultured at 37°C in a humidified atmosphere of 5% CO_2_. Two days after confluence, the cells were differentiated into adipocytes by changing media to Adipogenic Differentiation Media (ADM) (from Cellular Engineering Technologies, Inc., Coralville, IA) supplemented with 10% FBS. The medium was replaced with fresh ADM+10% FBS every 48 hours.

### Quantitative RT-PCR

Total RNA was extracted from cells using the TRIzol reagent (Life Technologies, Inc.) and transcribed into cDNA using Superscript II reverse transcriptase (Life Technologies, Inc.), random primer (Takara Bio, Inc., Shiga, Japan) and oligo (dT)12–18 (Life Technologies, Inc.). Quantitative PCR was conducted in 20 μl reactions containing first-strand cDNA template, SYBR GREEN PCR Master Mix (Applied Biosystems) and primer sets using ABI ViiA7 sequence detection system (Applied Biosystems). The following oligonucleotide primers for mouse T1R1, T1R2, T1R3, CaSR, Gαgust, Gα14, Gαs, β-actin and ribosomal protein S18 and human T1R2, T1R3 and β-actin were purchased from Takara Bio, Inc.: for mouse T1R1, 5′-GAGACACAGACCTCTGGTGACAA-3′ (forward) and 5′-CTGAGCACACGTCATACAGTTCATA-3′ (reverse); for mouse T1R2, 5′-CTGCTTCGAGTGTGTGGACTG-3′ (forward) and 5′-GAAGCAAGCGATGTTGTTCTTGTAA-3′ (reverse); for mouse T1R3, 5′-AAGGCCTGCAGTGCACAAGA-3′ (forward) and 5′-GGCCTTAGGTGGGCATAATAGGA-3′ (reverse); for mouse CaSR, 5′-TTTGGAGTAGCAGCCAAAGATCAAG-3′ (forward) and 5′-ACCATCGGAATCCACGGAAG-3′ (reverse); for mouse Gαgust, 5′-GCGGGATGCAAGAACTGTGA-3′ (forward) and 5′-ACTCCATGCATTCTTGTTTGCTGTA-3′ (reverse); for mouse Gα14, 5′-TGAACGACGGAAATGGATTCAC-3′ (forward) and 5′-ATGGTTCTAAACAGGGCTTTGCTC-3′ (reverse); for mouse Gαs, 5′-CATTCTGAGCGTGATGAACGTG-3′ (forward) and 5′-AGTCAATCAGCTGGTACTCATTGGA-3′ (reverse); and for mouse β-actin, 5′-CATCCGTAAAGACCTCTATGCCAAC-3′ (forward) and 5′-ATGGAGCCACCGATCCACA-3′ (reverse); for mouse S18, 5′-TTCTGGCCAACGGTCTAGACAAC-3′ (forward) and 5′-CCAGTGGTCTTGGTGTGCTGA-3′; for human T1R2, 5′-CCCTATGTCCATGTGTTCCAAGAG-3′ (forward) and 5′-CAGGCCTGGCATTCATATTCA-3′ (reverse); for human T1R3, 5′-GGGTTCCACTCCTGCTGCTA-3′ (forward) and 5′-AAGGTGCAGGCGATGTCGT-3′ (reverse); for human β-actin, 5′-TGGCACCCAGCACAATGAA-3′ (forward) and 5′-CTAAGTCATAGTCCGCCTAGAAGCA-3′ (reverse). Reaction mixtures were incubated for an initial denaturation at 95°C for 10 minutes, followed by 40 PCR cycles. Each cycle consisted of 95°C for 15 seconds and 60°C for 60 seconds. The mRNA levels of all genes were normalized using β-actin or ribosomal protein S18 as internal controls.

### Immunoblotting

For immunodetection of PPARγ, C/EBPα and aP2, cells were washed with PBS, lysed in Laemmli buffer, boiled and centrifuged for 10 min at 10,000 rpm at 4°C. The supernatant was subjected to SDS-PAGE and immunoblotting. For immunodetection of T1R3, cells were homogenized in PBS containing complete protease inhibitor cocktail (Roche), followed by centrifugation for 5 minutes at 7,500 rpm at 4°C. The supernatant was subjected to SDS-PAGE and immunoblotting. The blots were visualized by using Amersham ECL detection systems (GE Healthcare) and LAS-4000 luminescent image analyzer (GE Healthcare). The intensities of the bands were quantified by using Multi Gauge software (Fuji Photo Film, Tokyo). The protein amount was normalized with the amount of β-tubulin or actin as internal controls by either reprobing the each PVDF membrane or immunoblotting the same sample with anti-β-tubulin or anti-actin antibodies.

### Immunostaining

3T3-L1 cells differentiated on a cover slip were fixed with 3% (w/v) paraformaldehyde, and immunostained with anti-T1R3 and anti-GLUT4 primary antibodies and Alexa Fluor 568- or Alexa Fluor 488-conjugated secondary antibodies as described previously [17]. Cells were also stained with DAPI (4′,6-diamidino-2-phenylindole) to visualize the nuclei. Immunofluorescence images were obtained with FluoView FV1000 confocal microscope system (Olympus, Tokyo).

### Oil Red-O Staining

3T3-L1 cells or ATSCs at Day 6 of differentiation were washed twice with PBS and fixed in 3% (w/v) paraformaldehyde in PBS for 10 min at room temperature. After washing twice with PBS, cells were incubated with 60% isopropanol solution for 1 minute before staining with Oil Red-O solution (3 mg/mL 60% (v/v) isopropanol) for 20 min. Cells were washed once with 60% (v/v) isopropanol and twice with PBS before observation by microscopy. For quantification of the amount of Oil Red-O, the dye was extracted by incubation of the cells with 100% isopropanol for 20 min, and the absorbance at 518 nm was measured.

### Transfection of siRNA or Plasmid DNA

Small interfering RNA (siRNA) duplexes targeting for mouse Gαs (Table 1) were purchased as Dharmacon siGENOME SMARTpool from Thermo Fisher Scientific Inc. (Waltham, MA). 3T3-L1 preadipocytes grown on a 100 mm culture dish were dispersed with 0.05% trypsin in PBS. After washing three times with PBS, cells were resuspended in Electroporation Buffer (Bio-Rad). A 0.55 ml of cell aliquot was mixed with 0.2 ml of the mixture of four siRNA duplexes (5 nmole each) in a 0.4 cm-gap cuvette before single pulse of electroporation by using Gene Pulser Xcell (Bio-Rad) set at 200 V and 500 microfarads. Electroporated cells were resuspended in DMEM-HG containing 10% CS, seeded on a 12-well culture plate and cultured to confluence (usually for 2 days) before induction of differentiation as described above.

**Table 1:** Target Sequences for shRNA and siRNA.

Target	Gene symbol	Target sequence of mRNA
T1R3	Tas1r3	ACAUCACCAAUGCAAUGUU (2216–2234)
Gαs	Gnas1	GCUUAGAUGUUCCAAAUUU (1551–1569)
		GAUCAACACCGCAACCUUU (1486–1504)
		GGACUACUUUCCAGAGUUC (1134–1152)
		GAACAUCCGCCGUGUCUUC (1317–1336)

The pGIPz expression vectors containing short hairpin RNA (shRNA) targeting mouse T1R3 (Table 1) or non-silencing shRNA were purchased from Thermo Fisher Scientific Inc. (Waltham. MA). The expression plasmids (30 μg) were transfected into 3T3-L1 preadipocytes by electroporation, seeded on a 12-well culture plate and differentiated as described above.

The cDNA for wild-type rat G s was provided by Randall R. Reed [20] (Johns Hopkins University, Baltimore, MD) and subcloned into the pCMV5 expression vector. The cDNA construct for Gαs with a G226A mutation [21], [22] was prepared by using QuikChange II site-directed mutagenesis kit (Agilent Technologies). The expression plasmids (20 μg) for wild-type or G226A mutant Gαs were transfected into 3T3-L1 preadipocytes by electroporation, seeded on a 12-well culture plate and differentiated as described above.

The pcDNA3.1 expression vectors containing mouse T1R2 or T1R3 cDNA were provided by Yutaka Maruyama (Ajinomoto Company, Inc., Kawasaki, Japan). The expression plasmid (total of 20 μg) was transfected into HEK293 cells by electroporation as described above, seeded on a 12-well culture plate for the intracellular cAMP assay or on a 35 mm glass bottom culture dish for the real-time measurement of [cAMP]_c_ and cultured for 24 hours before assay.

### Measurement of Cellular cAMP Content

The cellular content of cAMP was measured by using AlphaScreen cAMP assay kit (PerkinElmer) according to the manufacturer's instruction. Briefly, cells on a 12-well culture plate were serum-starved for 3 hours and then incubated for 30 minutes at 37°C in Hanks' balanced salt solution (HBSS) containing 138 mM NaCl, 5.4 mM KCl, 1.3 mM CaCl_2_, 0.5 mM MgCl_2_, 0.38 mM MgSO_4_, 0.44 mM KH_2_PO_4_, 0.34 mM Na_2_HPO_4_, 5.5 mM D-glucose and 20 mM Hepes/NaOH, pH 7.4 before stimulation with sweetener in the presence of 0.5 mM IBMX for 30 minutes. At the end of incubation, 0.2 vol. of 0.5 M HCl was added and cells were lysed by freezing at −30°C and thawing before centrifugation at 15,000×g for 10 minutes at 4°C. The resultant supernatant was diluted with an equivalent volume of 200 mM Hepes/NaOH, pH 7.4 and used for assay.

### Real-Time Measurement of Cytosolic cAMP Concentration

The cytosolic cyclic AMP concentration ([cAMP]_c_) was measured by using Epac1-camp, a Epac-based cAMP sensor [23] as described previously with a slight modification [11]. Epac1-camp is comprised of a single cyclic nucleotide binding domain of Epac1 fused between enhanced cyan fluorescent protein (ECFP) and enhanced yellow fluorescent protein (EYFP). Binding of cAMP induces a conformational change leading to an increase in distance between the fluorophores, which is measured as a decrease in FRET (fluorescence resonance energy transfer). Thus, a decrease in the FRET emission ratio (EYFP/ECFP) is indicative of an increase in [cAMP]_c_. Briefly, cells were transfected with 30 μg of plasmid encoding Epac1-camp by electroporation as described above and seeded on a 35 mm glass bottom culture dish. After incubation for 24 hours, medium was removed and replaced with HBSS. For measurement of [cAMP]_c_, Epac1-camps was excited with the wavelength of 440 nm and dual emission images for ECFP and EYFP were obtained using AQUACOSMOS/ASHURA fluorescence resonance energy transfer imaging system (Hamamatsu Photonics, Hamamatsu, Japan). The [cAMP]_c_ data was presented as the reciprocal of the emission ratio of EYFP/ECFP (i.e. ECFP/EYFP).

### Statistical Analysis

Data was analyzed by Student's *t*-test and P<0.05 was considered as statistically significant.

## Results

**Figure 1:**
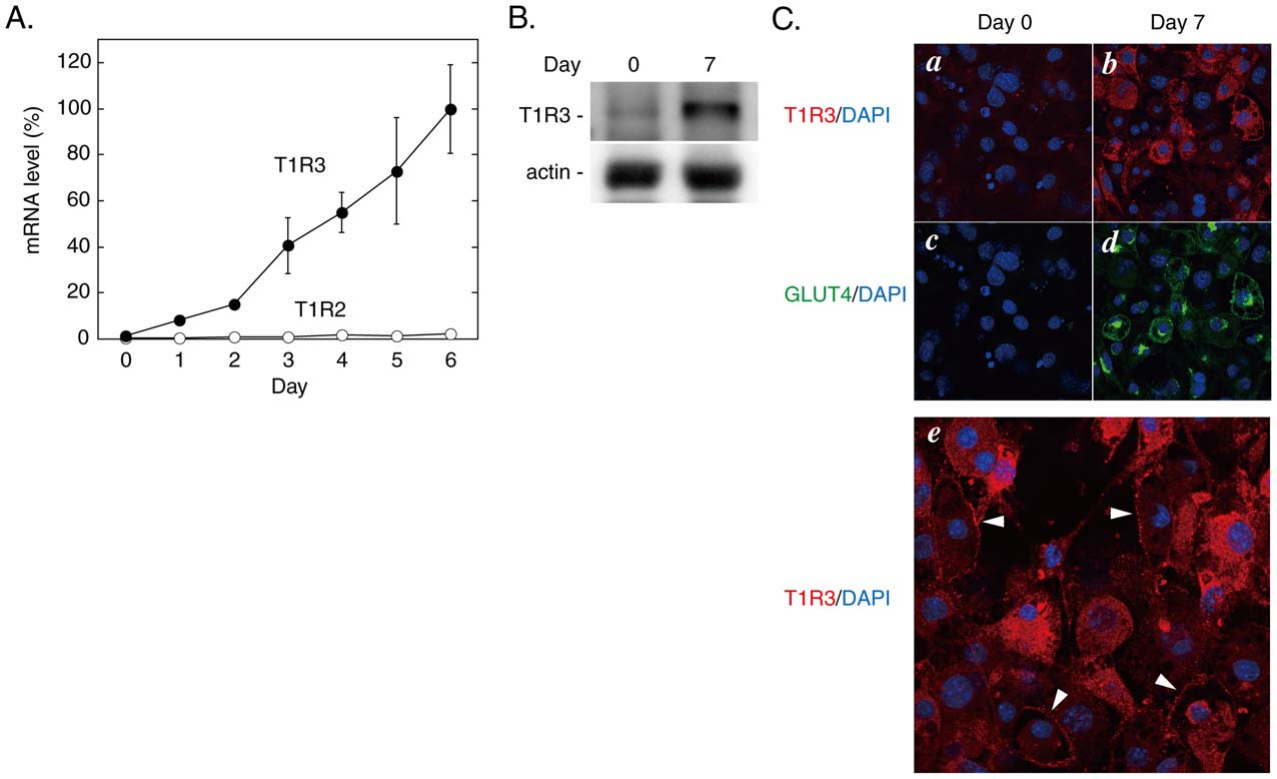
Expression of T1Rs during differentiation of 3T3-L1 cells. A. The total RNAs prepared from 3T3-L1 cells at the indicated time points during differentiation were subjected to quantitative RT-PCR using mouse ribosomal protein S18 as an internal control as described in`Materials and Methods'. The mRNA levels of T1R2 and T1R3 are shown as the percentage of that of T1R3 at Day 6. Results are shown as the mean ± SE (n = 3–6). B. Immumoblot data for T1R3 and actin in undifferentiated (Day 0) and differentiated (Day 7) 3T3-L1 cells. C. Immunofluorescence staining images for T1R3 (*a* and *b*, red) and GLUT4 (*c* and *d*, green) in undifferentiated (Day 0, *a* and *c*) and differentiated (Day 7, *b* and *d*) 3T3-L1 cells. Nuclei were visualized with DAPI (blue). *e*, Subcellular distribution of T1R3 (red) in Day 7 cells. Arrowheads indicate peripheral localization of T1R3.

We first examined by quantitative RT-PCR the expression profiles of T1R family GPCRs during the differentiation process of 3T3-L1 cells. As shown in Table 2, all the T1R GPCRs were very weakly expressed in 3T3-L1 preadipocytes compared with mouse circumvallate and foliate papillae. On the basis of the ratio to β-actin, the mRNA levels of T1R1, T1R2 and T1R3 in preadipocytes were 0.03, 0.01 and 0.6%, respectively, of that of T1R3 in the circumvallate papillae. The expression of T1R3, however, was dramatically up-regulated upon induction of differentiation (by 12.4-fold at Day 2 and by 83.0-fold at Day 6) whereas T1R2 showed a less prominent increase with differentiation (by 3.8-fold at Day 6) (Fig. 1A). Consequently, T1R3 was 17.7 and 46.2 times more abundant than T1R2 at Day 2 and Day 6, respectively. In agreement, immunoblotting data revealed a marked increase in the protein amount of T1R3 with differentiation (Fig. 1B). Immunofluorescence staining data also demonstrated an increase in T1R3 and GLUT4 proteins with differentiation and both proteins were considerably co-expressed in Day 7 cells (Fig. 1C, *a*–*d*). As shown with arrowheads in Fig. 1C (*e*), T1R3 was localized at the periphery in some but not all cells at Day 7. T1R3 signals were also present in the cytosol of Day 7 cells. These cytosolic signals may possibly represent immature T1R3 protein or mature T1R3 trapped by other interacting protein(s) in the internal compartment(s) although we cannot rule out another possibility that these signals are non-specific ones due to technical problems. Further studies with more sophisticated methods or more specific antibody will answer these points. To test whether these expression profiles in 3T3-L1 cells are physiologically relevant, we examined the expression levels of T1Rs in the mouse adipose tissue. As shown in Table 2, T1R2 and T1R3 were expressed in nearly equivalent levels in mouse circumvallate and foliate papillae. By contrast, a significantly high level of T1R3 was detected in epididymal adipocytes of C57BL/6J mouse, which was 420 times higher than T1R2. On the other hand, their expression levels in the stromal-vascular fraction (SVF) were comparable to those in 3T3-L1 preadipocyte. These results suggested that T1R3 would be expressed mainly as either a homomer or a heterodimer with another yet undefined receptor and that only a few T1R3 molecules would form a heterodimer with T1R2 in differentiating 3T3-L1 cells and mature adipocytes. In this regard, we did not detect the calcium-sensing receptor (CaSR), another member of the class C GPCR, which is expressed in taste buds and enhances sweet, salty, and umami tastes [24]–[26].

**Table 2:** Relative Expression Levels of the T1R Family GPCRs.

	circumvallate papillae	foliate papillae	SVF	adipocyte	3T3-L1 Day 0	3T3-L1 Day 6
T1R1	19.7	6.7	0.03	ND	0.03	2.40
T1R2	147.8	44.1	0.06	10.9	0.01	0.27
T1R3	100.0	54.2	1.58	4579.1	0.63	148.4

The mRNA levels of T1R family GPCRs in circumvallate and foliate papillae, stromal-vascular fraction (SVF) and adipocytes from epididymal fat pad of C57BL/6J mouse and 3T3-L1 preadipocytes (Day 0) and adipocytes (Day 6) were measured by quantitative RT-PCR as described in ‘Materials and Methods’. For comparison, the mRNA level of each gene was normalized as the ratio to β-actin mRNA, and results are shown as the percentage of the T1R3 mRNA level in circumvallate papillae. ND, not detected.

**Figure 2:**
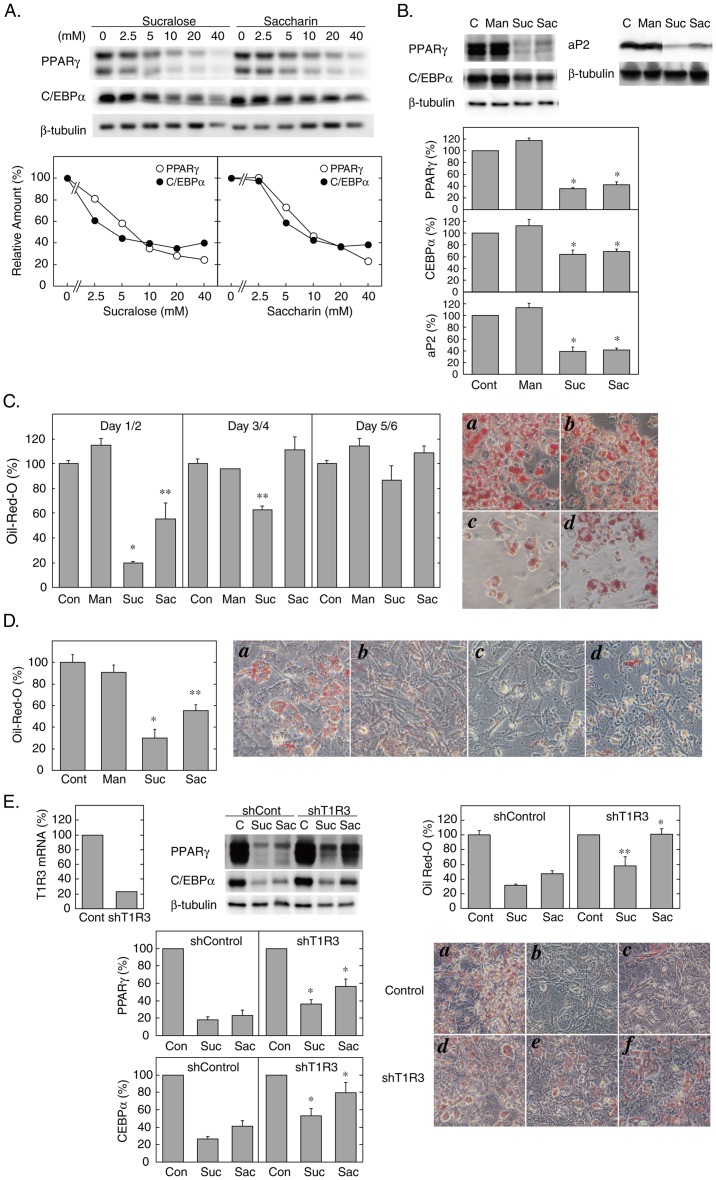
Effects of Sweeteners on Adipogenesis. A. 3T3-L1 cells were differentiated in the presence of the indicated concentrations of sucralose or saccharin, and the expression levels of PPARγ and C/EBPα at Day 2 (48 hours) were examined by immunoblotting. Representative immunoblot data for PPARγ and C/EBPα (upper panel) and the relative amounts of the proteins normalized with β-tubulin (lower panel) are shown. Results are shown as the mean values of two independent experiments. B. 3T3-L1 cells were differentiated without (control) or with D-mannitol (20 mM), sucralose (20 mM), or saccharin (20 mM) during the first 48 hours of differentiation. The expression levels of PPARγ and C/EBPα at Day 2, and aP2 at Day 4 were examined by immunoblotting. Representative immunoblot data (upper panel) and the relative amounts of the proteins normalized with β-tubulin (lower panel) are shown. Results are shown as the mean ± SE (n = 3). ?, P<0.05 (vs. Control). C. 3T3-L1 cells were differentiated in the absence (control) or the presence of D-mannitol (20 mM), sucralose (20 mM), or saccharin (20 mM) during the first 48 hours (Day 1 and 2), the second 48 hours (Day 3 and 4) or the third 48 hours (Day 5 and 6) of differentiation, and were stained with Oil Red-O at Day 6. The amounts of dye were quantified as described in ‘Materials and Methods’ (left panel). Results are shown as the mean ± SE (n = 3). ?, P<0.005; ??, P<0.05 (vs. Control). Con, control; Man, D-mannitol; Suc, sucralose; Sac, saccharin. Microscopic images of Oil Red-O stained cells differentiated with the addition of none (control) (*a*), D-mannitol (20 mM) (*b*), sucralose (20 mM) (*c*) or saccharin (20 mM) (*d*) during the first 48 hours are shown (right panel). D. Adipose tissue-derived stem cells were differentiated to adipocytes as described in ‘Materials and Methods’ in the presence of none (control) (*a*), D-mannitol (20 mM) (*b*), sucralose (20 mM) (*c*) or saccharin (20 mM) (*d*) for 6 days, and were stained with Oil Red-O. The amounts of Oil Red-O (left panel) and microscopic images (right panel) are shown. Results are presented as the mean ± SE (n = 3). ?, P<0.001; ??, P<0.01 (vs. control). E. Undifferentiated 3T3-L1 cells were transfected with the pGIPz expression vectors containing non-silencing or T1R3-targeting shRNA sequences (30 μg each) by electroporation as described in ‘Materials and Methods’. Transfected cells were seeded on a 12-well dish and cultured to confluence before induction of differentiation without (control) or with sucralose (20 mM) or saccharin (20 mM). The expression level of T1R3 was measured by quantitative RT-PCR immediately before induction of differentiation (at Day 0) (left panel). The expression levels of PPARγ and C/EBPα at Day 2 (48 hours) were measured by immunoblotting. Representative immunoblot data and the relative amounts of the proteins normalized with β-tubulin are shown (middle panel). Results are presented as the mean ± SE (n = 3). ?, P<0.05 (vs. shControl). The amounts of Oil Red-O and the microscopic images of stained cells at Day 6 are shown (right panel). Cells transfected with non-silencing (*a*–*c*) or T1R3-targeting shRNA (*d*-*f*) were differentiated in the absence (*a* and *d*) or the presence of sucralose (20 mM) (*b* and *e*) or saccharin (20 mM) (*c* and *f*) during the first 48 hours. Results are presented as the mean ± SE (n = 3). ?, P<0.01; ??, P<0.05 (vs. shControl).

Next, we examined whether these T1Rs would function as a receptor for sweet compounds in 3T3-L1 cells, and if so, what type of signaling mechanisms it would employ. As shown in Fig. 2A, the addition of sucralose or saccharin during the first 48 hours of differentiation inhibited the expression of peroxisome proliferator activated receptor γ (PPARγ and CCAAT/enhancer-binding protein α (C/EBPα at Day 2 in a dose-dependent manner with EC_50_s in the mM range. In addition, sucralose and saccharin also inhibited the expression of aP2 at Day 4 (Fig. 2B) and reduced the accumulation of triglyceride at Day 6 (Fig. 2C). By contrast, the addition of the sweeteners during the second 48 hours (Days 3 and 4) or the third 48 hours (Days 5 and 6) of the differentiation process little affected the accumulation of triglyceride at Day 6 except that sucralose showed a significant inhibition when added during Days 3 and 4 (Fig. 2C, left panel). These data indicated that 3T3-L1 cells express a functional sweet taste-sensing receptor, which negatively regulates adipogenic differentiation. To test the physiological relevance of these findings in 3T3-L1 cells, we also examined the effects of sucralose and saccharin on the differentiation of primary adipose tissue-derived stromal cells (ATSCs) from C57BL/6J mice. As shown in Fig. 2D, the addition of sweeteners in the differentiation media significantly inhibited triglyceride accumulation in ATSCs at Day 6, consistent with the notion that sweetener would inhibit differentiation of primary adipocytes.

These findings raised a possibility that either a homomer of T1R3 or a heterodimer of T1R3 and another yet undefined GPCR would function as a sweet taste-sensing receptor in 3T3-L1 cells. To clarify the role for T1R3 in mediating the anti-adipogenic effects, we reduced T1R3 expression by short hairpin RNA (shRNA)-mediated gene silencing. As depicted in Fig. 2E, transfection of plasmids containing the shRNA sequences targeting T1R3 efficiently decreased T1R3 expression by ∼80% and significantly interfered with sweetener-induced inhibition of PPARγ and C/EBPα at Day2 and triglyceride accumulation at Day 6. These data suggested that T1R3 is involved in mediating the anti-adipogenic effect of sweeteners.

**Figure 3:**
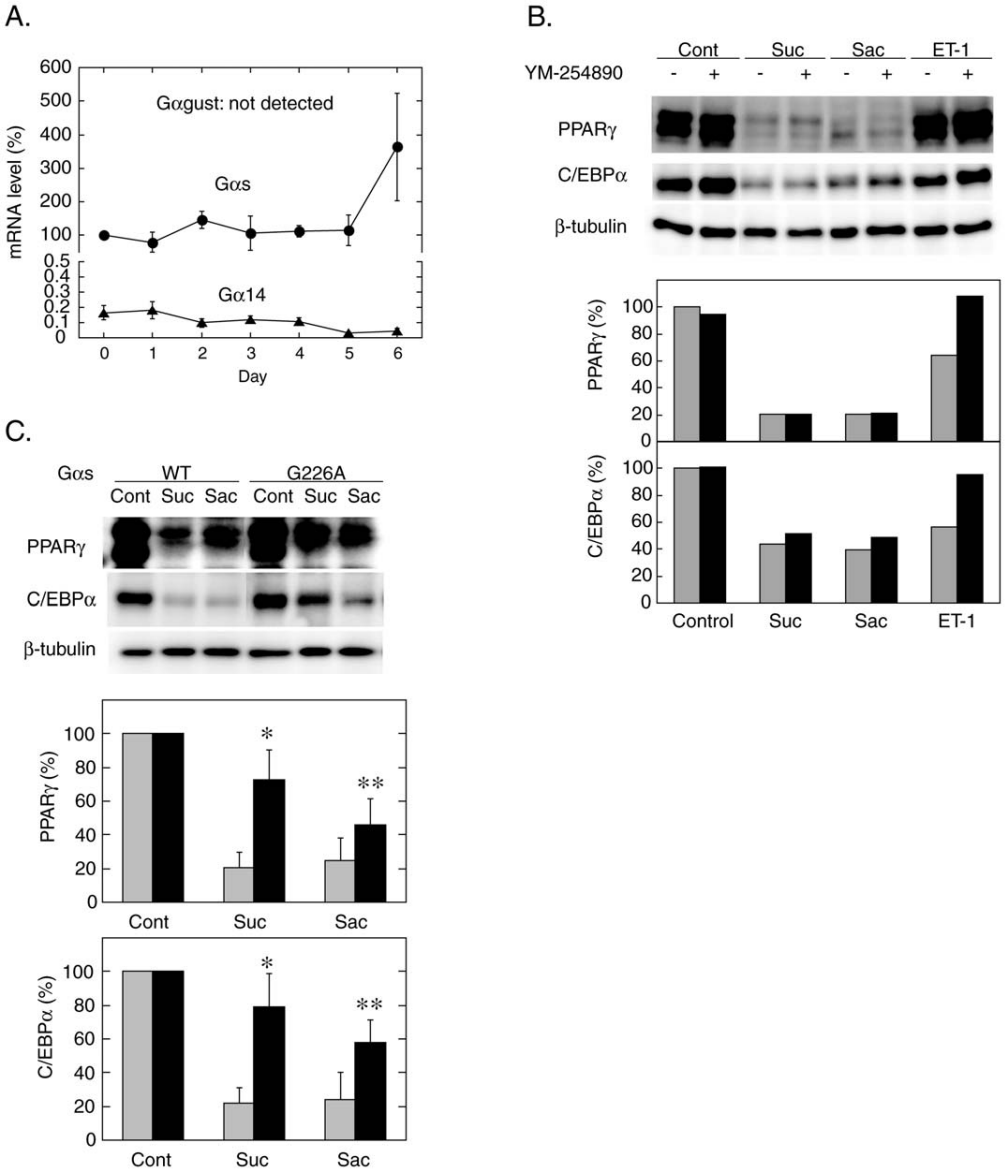
Roles for G proteins in Sweeteners Effects on Differentiation of 3T3-L1 cells. A. Expression profiles of Gαgust, Gα14 and Gαs during differentiation of 3T3-L1 cells. The total RNAs were prepared from 3T3-L1 cells as described in Fig. 1 and the mRNA levels of Gαgust, Gα14 and Gαs were measured by quantitative RT-PCR using mouse ribosomal protein S18 as an internal control. Results are shown as the mean ± SE (n = 3–6). B. 3T3-L1 cells were differentiated without (control) or with sucralose (20 mM), saccharin (20 mM), or endothelin-1 (20 nM) in the absence (0.1% DMSO) or the presence of YM-254890 (10 µM). The expression levels of PPARγ and C/EBPα at Day 2 (48 hours) were measured by immunoblotting. Representative immunoblot data (upper panel) and the relative amounts of the proteins normalized with β-tubulin (lower panel) are shown. Gray and black bars show the control and the plus YM-254890 data, respectively. Results are shown as the mean values from two independent experiments. C. Undifferentiated 3T3-L1 cells were detached and transfected with the expression vectors containing wild-type or G226A mutant Gαs cDNAs (20 μg each) by electroporation as described in ‘Materials and Methods’. Transfected cells were seeded on a 6-well culture dish and cultured to confluence before induction of differentiation without (control) or with sucralose (20 mM) or saccharin (20 mM). The expression levels of PPARγ and C/EBPα were measured by immunoblotting at Day 2 (48 hours). Representative immunoblot data (upper panel) and the relative amounts of the proteins normalized with β-tubulin (lower panel) are shown. Gray and black bars show the control and the Gαs-G226A data, respectively. Results are shown as the mean ± SE (n = 3). P<0.01; P<0.05 (vs. wild-type).

To explore the signaling mechanism downstream of the putative sweet taste-sensing receptor, we next examined the expression of the α-subunits of trimeric G proteins that might be coupled with the sweet taste receptor: gustducin (Gαgust), G14 (Gα14) and Gs (Gαs). As described earlier, gustducin has been accepted as the sweet taste receptor-coupled G protein. G14, a member of the Gq family G proteins, is also co-expressed with T1R2 and T1R3 in taste receptor cells especially in the posterior portion of the tongue and is thought as another candidate for the sweet taste receptor-coupled G protein [27], [28]. Although the role for Gs in sweet taste response is yet to be fully defined, previous studies have shown that Gαs and several isoforms of adenylate cyclase are expressed in taste buds [29], [30] and that sucrose and saccharin activate adenylate cyclase with elevation of the cAMP concentration in taste buds [30]–[33]. Additionally, we have recently reported that stimulation with sucralose or saccharin of mouse insulinoma MIN6 cells caused an elevation of the cytosolic cAMP concentration [11] and this effect was attenuated by siRNA-mediated knockdown of Gαs (unpublished observation by Y. Nakagawa and I. Kojima), suggesting the possible coupling of the sweet taste receptor with Gs. As shown in Fig. 3A, quantitative RT-PCR analyses demonstrated that Gαgust was not detected throughout the differentiation process, while both Gα14 and Gαs were continuously expressed although the expression level of Gα14 remained less than 0.2% of Gαs.

To define the G protein mediating the anti-adipogenic signal downstream of the sweet taste-sensing receptor in 3T3-L1 cells, we firstly interfered with the function of G14 by using a pharmacological inhibitor. As shown in Fig. 3B, the addition of YM-254890, a specific inhibitor of Gq family G proteins such as Gq, G11 and G14 [34], [35], in the media canceled the inhibition with endothelin-1 of PPARγ and C/EBPα expression at Day 2 but not the effects of sucralose and saccharin. These data suggested that endothelin-1 inhibits differentiation of 3T3-L1 cells through activation of a YM-254890-sensitive G protein, probably Gq [36]–[40], whereas none of those Gq family proteins including G14 mediate the anti-adipogenic signal from the sweet taste-sensing receptor. On the other hand, overexpression of the dominant-negative mutant of Gαs (Gαs-G226A) [21], [22] markedly attenuated the inhibitory effects of sweeteners on PPARγ and C/EBPα (Fig. 3C), suggesting that the anti-adipogenic signal of sweeteners would be mediated via Gs.

**Figure 4:**
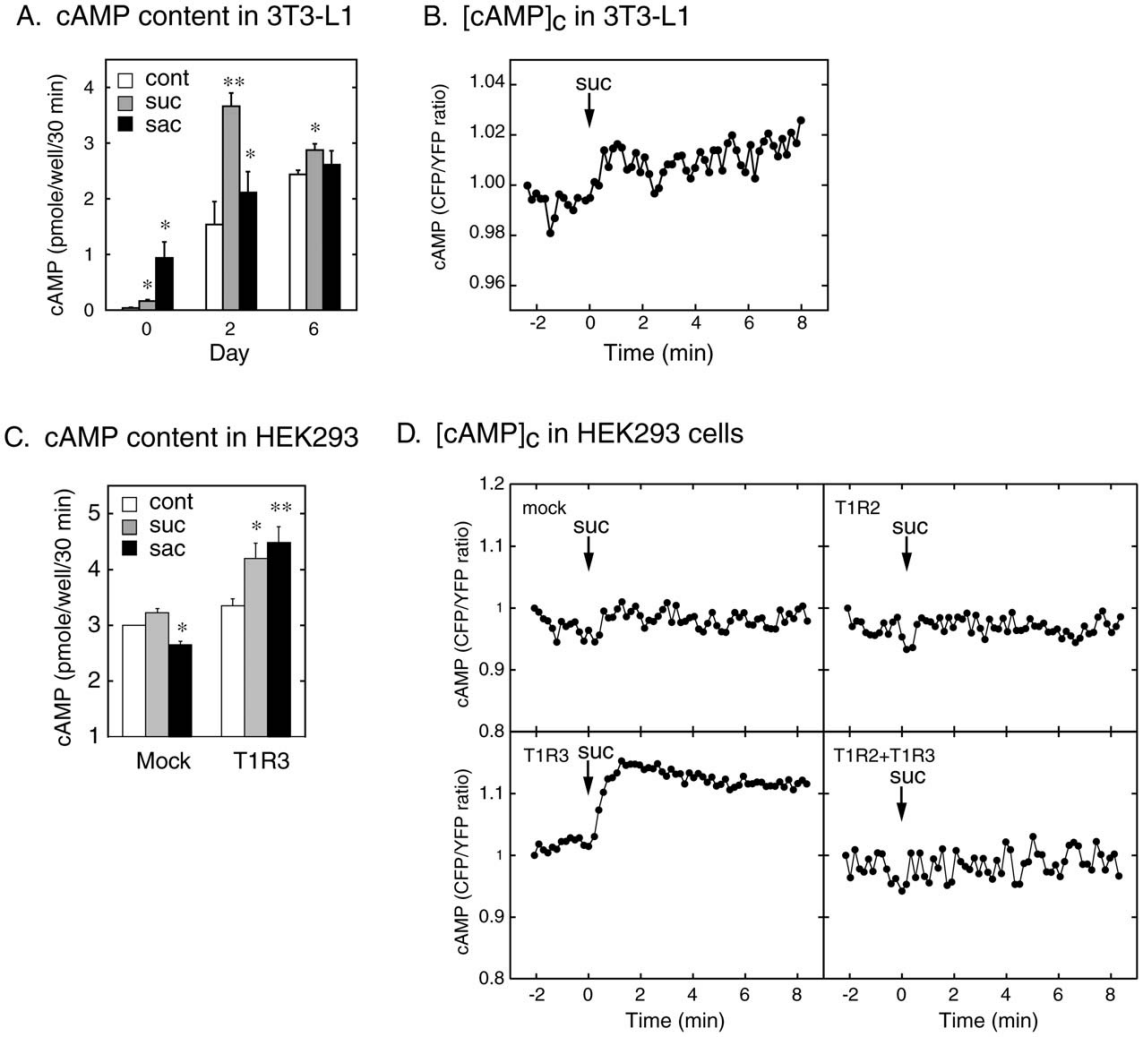
Effects of Sweeteners on cAMP. A. 3T3-L1 cells at Day 0 (undifferentiated), Day 2 and Day 6 of differentiation were stimulated without (control) or with sucralose (20 mM) or saccharin (20 mM) for 30 min at 37°C, and the cellular cAMP contents were measured as described in ‘Materials and Methods’. Results are shown as the mean ± SE (n = 3)., P<0.05, P<0.01 (vs. control). B. 3T3-L1 cells at Day 6 of differentiation were transfected with 30 μg of the expression plasmid encoding Epac1-camps cDNA by electroporation, seeded on a 35 mm glass bottom dish and cultured for 24 hours before the measurement of the cytosolic cAMP concentrations ([cAMP]_c_). At the time point indicated with the arrow, cells were stimulated with 20 mM of sucralose. The [cAMP]_c_ was shown as the reciprocal of the emission ratio of EYFP/ECFP (i.e. ECFP/EYFP). C. HEK293 cells were transfected with pcDNA3.1 vector (20 μg) alone (mock) or with the vector containing mouse T1R3 cDNA (20 μg) by electroporation and seeded on a 12-well culture plate. After incubation for 24 hours, cells were stimulated without (control) or with sucralose (20 mM) or saccharin (20 mM) for 30 minutes at 37°C and the cellular contents of cAMP were measured as described in ‘Materials and Methods’. Results are shown as the mean ± SE (n = 4–6)., P<0.05, P<0.01 (vs. control). D. HEK293 cells transfected by electroporation with pcDNA3.1 vector (mock), T1R2 (20 μg), T1R3 (20 μg), or both T1R2 and T1R3 (10 μg, each) were seeded on a 35 mm glass bottom dish, cultured for 24 hours and stimulated with 20 mM of sucralose at the time point indicated with the arrow. The [cAMP]_c_ were monitored as described in B.

This led us to examine whether the putative sweet taste-sensing receptor is coupled with Gs in 3T3-L1 cells. As shown in Fig. 4A, sucralose and saccharin increased the cAMP content in differentiating 3T3-L1 cells at Day 2 and Day 6. The real-time measurement of [cAMP]_c_ in 3T3-L1 cells also demonstrated a rapid increase in [cAMP]_c_ with sucralose stimulation (Fig. 4B). These results suggested that the putative sweet-sensing receptor is likely coupled with Gs and activates adenylate cyclase. The effects on cAMP of the sweeteners, however, were smaller at Day 6 than Day 2 despite the higher expression level of T1R3 (Fig. 4A). The reason for this is unclear, but it is possible that the increase in the expression level of T1R3 itself activated adenylate cyclase and blunted the effect of sweeteners [41]. It is also possible that the tonic inhibition of adenylate cyclase with endogenous adenosine modulated the effects of sweeteners [42]. Further study would be necessary to clarify these points.

To attempt to identify the molecular entity of the sweet taste-sensing receptor in 3T3-L1 cells, we examined the cAMP response to sweet compounds in HEK293 cells heterologously expressing mouse T1R3. As depicted in Fig. 4C, sucralose and saccharin increased the cellular cAMP content in HEK293 cells transfected with T1R3 alone. In the real-time measurement of [cAMP]_c_, sucralose also increased [cAMP]_c_ in T1R3-expressing HEK293 cells, but not in cells transfected with T1R2 alone or with both T1R2 and T1R3 (Fig. 4D). Since endogenous T1R2 and T1R3 were expressed at negligible levels in HEK293 cells (1.40±0.49×10^−6^ and 22.5±6.6×10^−6^, respectively, as the ratio to the actin mRNA), which were less than 10% of those in 3T3-L1 preadipocytes, it was unlikely that their expression would have affected the cAMP response in the overexpression study. These data supported the notion that a homomer of mouse T1R3 could function as a sweet taste-sensing receptor that elevates cAMP at least in HEK293 cells.

**Figure 5:**
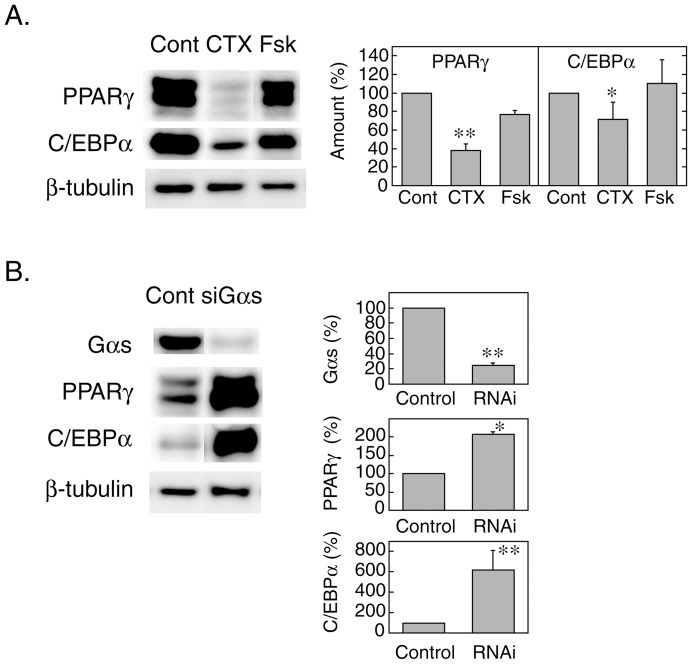
Role for Gαs in adipogenesis. A. 3T3-L1 cells were differentiated for 48 hours in the absence (control) or the presence of cholera toxin (0.1 μg/ml) or forskolin (20 μM). Then the expression levels of PPARγ and C/EBPα were measured by immunoblotting. The representative immunoblot data (left panel) and the relative amounts of the proteins normalized with β-tubulin (right panel) are shown. Data are shown as the mean ± SE (n = 3)., P<0.05, P<0.01 (vs. control). Cont, control; CTX, cholera toxin; Fsk, forskolin. B. Undifferentiated 3T3-L1 cells were transfected with 20 nmoles of non-silencing or Gαs-targeting siRNAs by electroporation as described in ‘Materials and Methods’. Cells were seeded on a 12-well plate, cultured to confluence and differentiated for 48 hours before the measurement of expression levels of PPARγ and C/EBPα by immunoblotting. The representative immunoblot data (left panel) and the relative amounts of the proteins normalized with β-tubulin (right panel) are shown. Data are shown as the mean ± SE (n = 3)., P<0.05, P<0.01 (vs. control).

It seemed unlikely, however, that Gs-mediated activation of adenylate cyclase with elevation of cAMP is essential for the inhibition of adipogenesis, since cAMP-dependent processes are pivotal during the early stages of adipocyte differentiation [43], [44]. In this regard, previous observations by other investigators [45]–[48], demonstrated the negative regulatory role of Gs in adipogenesis. We thus investigated the role for Gs in adipogenic differentiation of 3T3-L1 cells. Activation of Gs with cholera toxin inhibited the expression of PPARγ and C/EBPα at Day 2, while direct activation of adenylate cyclase with forskolin showed insignificant inhibitory effects on the expression of the transcription factors (Fig. 5A). By contrast, siRNA-mediated knockdown of Gαs markedly up-regulated the expression of PPARγ and C/EBPα at Day 2 (Fig. 5B). These results were in good agreement with previous observations and confirmed the negative regulatory role of Gs in adipogenesis.

## Discussion

**Figure 6:**
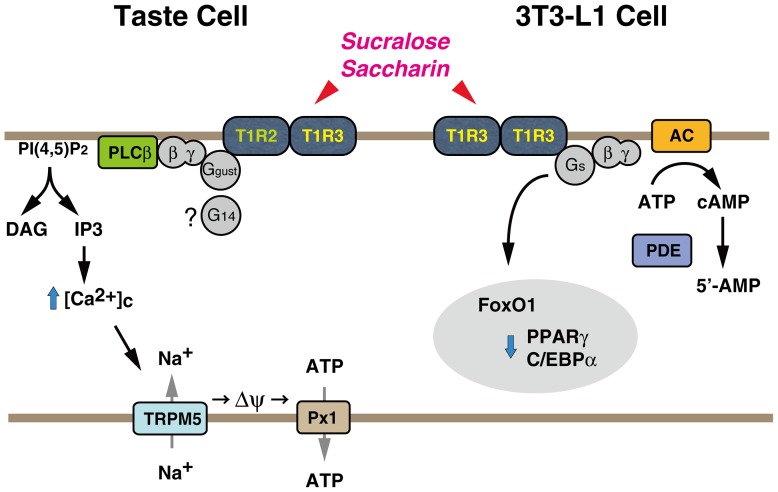
A model for signal transduction mechanism downstream of the sweet taste-sensing receptors in taste cells and 3T3-L1 cells. In taste cells (in the left side), T1R2+T1R3 heterodimeric sweet receptor activates PLCβ via gustducin (Ggust) or other G proteins, leading to [Ca^2+^]_c_ elevation and membrane depolarization. In 3T3-L1 cells (in the right side), T1R3 homomeric receptor may activates Gs, which mediates the anti-adipogenic signal by a cAMP-independent mechanism. PLCβ: phospholipase C-β; DAG: diacylglycerol; IP_3_: inositol 1,4,5-trisphosphate; Px1: pannexin 1; AC: adenylate cyclase; PDE: cAMP phosphodiesterase.

In the present study, we demonstrated the expression of a sweet-taste sensing receptor in differentiating adipocytes, which plays a negative regulatory role in adipogenic differentiation of 3T3-L1 cells, thus providing evidence for a novel nongustatory function of the sweet taste-sensing receptor. The molecular entity of the sweet taste-sensing receptor expressed in 3T3-L1 cells, however, would be distinct from the well-known T1R2+T1R3 heterodimer expressed in taste buds, since the expression levels of T1R2 and T1R3 were not equivalent but the latter was 17.7 and 46.2 times more abundant at Day 2 and Day 6, respectively, than the former (Fig. 1A). Quantitative RT-PCR analyses also demonstrated that T1R3 was expressed at a significantly higher level than T1R2 in mature adipocytes, whereas their expression levels in SVF were as low as in 3T3-L1 preadipocytes (Table 2). This indicated that the majority of T1R3 are expressed as a homomer or a heterodimer with another undefined GPCR in differentiating and mature adipocytes. Nelson et al. [1] predicted the existence of such a non-canonical taste receptor from their morphological observations that a fraction of taste cells express T1R3 but neither T1R1 nor T1R2. The same group also demonstrated that, with co-expression of gustducin, T1R3-expressing HEK293 cells showed an elevation of [Ca^2+^]_c_ in response to high concentrations of natural sugars [5]. By contrast, our heterologous expression study showed that T1R3 alone could function as a sweet taste-sensing receptor that increases cAMP with sweet stimuli in HEK293 cells (Fig. 4). Thus, in contrast to the T1R2+T1R3 heterodimer, the non-canonical T1R3 homomer could activate the adenylate cyclase-cAMP signaling pathway in the absence of gustducin (see Figure 6 for our working model).

The present study also demonstrated that the putative T1R3 homomeric receptor is likely coupled with Gs but none of YM-254890-sensitive Gq family proteins including G14, and mediates the anti-adipogenic signal through activation of Gαs in 3T3-L1 cells. Firstly, stimulation with sucralose or saccharin caused an elevation of cAMP in differentiating 3T3-L1 cells as well as in HEK293 cells expressing mouse T1R3 (Fig. 4). Secondly, overexpression of the dominant negative mutant of Gαs significantly attenuated the inhibitory effects of sweeteners on PPARγ and C/EBPα expression (Fig. 3). Thirdly, Gαs activation with cholera toxin treatment mimicked the anti-adipogenic effects of sucralose and saccharin, whereas siRNA-mediated knockdown of Gαs enhanced the expression of these adipogenic transcription factors (Fig. 5). Nevertheless, it was unlikely that the anti-adipogenic signal of the receptor is mediated by cAMP since forskolin, a direct activator of adenylate cyclase, did not inhibit the expression of PPARγ and C/EBPα (Fig. 5).

While these findings are consistent with previous observations [45]–[48] that demonstrated the anti-adipogenic role of Gαs, the downstream effector remains obscure at present. In agreement with the present study, Zhang et al. [48] have shown that overexpression of constitutively active mutants of TSH receptor or Gαs prevented adipogenesis of 3T3-L1 cells despite increased cAMP and CREB phosphorylation. The authors demonstrated that constitutively active Gαs reduced PPARγ expression through inhibition of FoxO1 phosphorylation by repressing transcription of WD repeat and FYVE domain-containing protein 2 (WDFY2), which facilitates FoxO1 phosphorylation by Akt through binding to phospho-Akt, although the direct downstream effector of Gαs has remained to be identified. Over the past decade, several non-canonical roles of heterotrimeric G proteins have been reported [49]. These include Gαs-mediated destabilization of the microtubules via promotion of tubulin GTPase [50] and EGF receptor degradation via Gαs interaction with hepatocyte growth factor-regulated tyrosine kinase substrate (Hrs), a critical component of the endosomal sorting machinery [51]. Taking into consideration that a certain type of Rho-GEF (*e.g*. GEF-H1) is activated by disassembly of the microtubules [52] and that Rho is a negative regulator in adipogenesis [53], it is intriguing to investigate whether sweetener stimulation may cause disassembly of the microtubules and activation of the Rho-mediated signaling pathway. This possibility is currently under investigation.

Finally, the present data also give insights into the molecular entity of the sweet taste receptor in taste buds that elevates cAMP. With regard to the intracellular signals generated by activation of the sweet taste receptor, both cAMP and calcium may act as second messengers. As described earlier, several studies have suggested involvement of Gs and adenylate cyclase in the signaling pathway downstream of the sweet taste receptor, whereas the molecular entity of the cAMP-elevating sweet taste receptor has remained obscure. On the other hand, a T1R3 homomer has been assumed as the possible sweet taste receptor [1], [5], whereas it has remained still open whether a T1R3 homomer might be the putative cAMP-elevating receptor. Our heterologous expression study demonstrated that this would be the case in HEK293 cells overexpressing mouse T1R3. Thus, it is interesting to investigate the possibility that a T1R3 homomer would function as the cAMP-elevating sweet taste receptor in taste receptor cells.

Our present working model for signal transduction mechanisms downstream of the sweet taste-sensing receptor in 3T3-L1 cells is diagramed in Figure 6. In taste cells (in the left side), T1R2+T1R3 heterodimeric sweet receptor activates PLCβ via gustducin (Ggust) or other G proteins, leading to [Ca^2+^]_c_ elevation and membrane depolarization (see ‘Introduction’ for details). In 3T3-L1 cells (in the right side), T1R3 homomeric receptor may be coupled with Gs, which generates both adenylate cyclase/cAMP-dependent pro-adipogenic and cAMP-independent anti-adipogenic signals. The latter signal may dominate over the former signal by unknown mechanisms and inhibits adipogenesis during the early stages of differentiation.

In summary, 3T3-L1 cells express a unique sweet taste-sensing receptor, which is distinct from the well-known T1R2+T1R3 heterodimeric receptor expressed in taste buds and may activate a different signaling pathway irrelevant to PLCβ and [Ca^2+^]_c_. This non-canonical sweet taste-sensing receptor may negatively regulate adipogenesis by a Gs-dependent but cAMP-independent mechanism. Further studies such as adipocyte-specific knockout of T1R3 are needed to explore the precise physiological relevance of this receptor. This unique sweet taste-sensing receptor may possibly be a target for treatment of obesity-related diseases such as type 2 diabetes and metabolic syndrome.
